# Optimal Target Level of Low-density Lipoprotein Cholesterol for Vascular Function in Statin Naïve Individuals

**DOI:** 10.1038/s41598-017-09043-1

**Published:** 2017-08-21

**Authors:** Shogo Matsui, Masato Kajikawa, Eisuke Hida, Tatsuya Maruhashi, Yumiko Iwamoto, Akimichi Iwamoto, Nozomu Oda, Shinji Kishimoto, Takayuki Hidaka, Yasuki Kihara, Kazuaki Chayama, Chikara Goto, Yoshiki Aibara, Ayumu Nakashima, Farina Binti Mohamad Yusoff, Kensuke Noma, Yukihito Higashi

**Affiliations:** 1 Department of Cardiovascular Medicine, Hiroshima University Graduate School of Biomedical Sciences, Hiroshima, Japan; 2 Division of Regeneration and Medicine, Medical Center for Translational and Clinical Research, Hiroshima University Hospital, Hiroshima, Japan; 3 Center for Integrated Medical Research, Hiroshima University Hospital, Hiroshima, Japan; 4 Department of Gastroenterology and Metabolism, Institute of Biomedical and Health Sciences, Graduate School of Biomedical and Health Sciences, Hiroshima University, Hiroshima, Japan; 5 Hiroshima International University, Hiroshima, Japan; 6 Department of Cardiovascular Regeneration and Medicine, Research Institute for Radiation Biology and Medicine, Hiroshima University, Hiroshima, Japan

## Abstract

We investigated (1) the relationship between low-density lipoprotein cholesterol (LDL-C) and vascular function in patients receiving and those not receiving statin therapy and (2) optimal level of LDL-C for maintenance of vascular function. Flow-mediated vasodilation (FMD) and nitroglycerine-induced vasodilation (NID) were inversely correlated with LDL-C in the 957 statin naïve subjects but not in the 392 subjects receiving statin therapy. In statin naïve subjects, non-high LDL-C (≤100 mg/dL) was independently associated with a decrease in adjusted odds ratio of the low tertile of FMD (OR: 0.62, 95% CI: 0.45–0.85; P = 0.003) and NID (OR: 0.69, 95% CI: 0.50–0.96; P = 0.03). Adjusted odds ratio of the low tertile of FMD was significantly lower in the low LDL-C group (≤70 mg/dL) (OR: 0.47, 95% CI, 0.27–0.81; P = 0.006) and in the moderate LDL-C group (70.1–100 mg/dL) (OR: 0.66, 95% CI, 0.48–0.94; P = 0.02) than in the high LDL-C group (>100 mg/dL). There was no significant difference in FMD between the low LDL-C group and moderate LDL-C group. There were significant relationships of FMD and NID with LDL-C levels in statin naïve subjects. In a general population, LDL-C of ≤100 mg/dL may be the optimal target level for maintenance of endothelial function.

## Introduction

Current evidence indicates that dyslipidemia, including a high level of low-density lipoprotein cholesterol (LDL-C), is a strong predictor of cardiovascular disease^1–3^. Lowering LDL-C by statin therapy or non-statin therapy [e.g., ezetimibe and proprotein convertase subtilisin/kexin type 9 (PCSK9)] reduces major cardiovascular events^4–6^. Several clinical guidelines, but not all of the guidelines, emphasize the importance of achievement of a targeted level of LDL-C by statin therapy^7–11^. However, the optimal target levels of LDL-C to prevent cardiovascular diseases are different in the guidelines^7–11^.

Endothelial dysfunction is an initial step of atherosclerosis, leading to cardiovascular diseases^12, 13^. Measurement of flow-mediated vasodilation (FMD) of the brachial artery as an index of endothelium-dependent vasodilation is a useful method for assessing endothelial function and is an independent predictor of cardiovascular diseases^14–23^. Moreover, several lines of evidence suggest that interventions, including pharmacological therapy and life style modification, improve endothelial function^24–29^.

Nitroglycerine-induced vasodilation (NID), an index of endothelium-independent vasodilation, assessed by sublingual administration of a nitroglycerine tablet is usually measured as a control test for FMD measurement to differentiate endothelium-dependent vasodilation from endothelium-independent vasodilation since both endogenous nitric oxide (NO) and administered nitroglycerine act on vascular smooth muscle cells^15, 30–32^. We previously reported that NID as well as FMD significantly correlated with cardiovascular risk factors^33^. In addition, the combination of measurements of FMD and NID is useful for predicting future cardiovascular events^34^.

Although it is established that endothelial function assessed by FMD and NID significantly correlate with conventional cardiovascular risk factors, it remains controversial whether elevation of serum levels of LDL-C is independently associated with endothelial dysfunction^21, 35, 36^. Although the reason for the controversial relationship between LDL-C levels and vascular function is unclear, we hypothesized that the association of LDL-C levels with vascular function might disappear under the influence of cholesterol-lowering therapy, especially statin therapy. In addition, the cutoff level of LDL-C for vascular dysfunction is not determined.

The aims of this study were (1) to determine the association of LDL-C with vascular function in subjects receiving and those not receiving statin therapy, and (2) to determine the optimal cutoff level of LDL-C for maintenance of endothelial function.

## Results

### Baseline characteristics

Baseline characteristics of all subjects are summarized in Table 1. Of the 1349 subjects, 787 (58.3%) were men and 562 (41.7%) were women, and 162 (12.0%) had coronary artery disease, 86 (6.4%) had stroke, and 392 (29.1%) were receiving statin therapy. We divided the subjects into 4 groups according to the statin use and level of LDL-C. Baseline characteristics of each group are shown in Table 1. In statin naïve subjects, there were significant differences between the non-high LDL-C (≤100 mg/dL) group and high LDL-C (>100 mg/dL) group in age, gender, body mass index, systolic blood pressure, diastolic blood pressure, total cholesterol, high-density lipoprotein cholesterol, hemoglobin A1c, and prevalence of hypertension and current smoking. FMD and NID were inversely correlated with age, body mass index, systolic blood pressure, diastolic blood pressure, glucose, hemoglobin A1c, and pack years of smoking (Supplementary Table 1). FMD, but not NID, was inversely correlated with total cholesterol and triglycerides.

**Table 1:** Clinical Characteristics of the Subjects in Accordance with LDL-C.

Variables	Total (1349)	Subjects not receiving statin therapy			Subjects receiving statin therapy		
		LDL-C≤100 (n = 400)	LDL-C>100 (n = 557)	P value	LDL-C≤100 (n = 251)	LDL-C>100 (n = 141)	P value
Age, yr	59 ± 17	52 ± 20	57 ± 16	< 0.001	69 ± 9	67 ± 11	0.07
Gender, men/women	787/562	279/121	306/251	< 0.001	140/111	62/79	0.62
Body mass index, kg/m^2^	23.7 ± 3.9	22.6 ± 3.6	24.1 ± 4.1	< 0.001	24.5 ± 4.0	24.3 ± 3.2	0.66
Systolic blood pressure, mmHg	131 ± 19	127 ± 20	134 ± 20	< 0.001	133 ± 19	133 ± 18.	0.93
Diastolic blood pressure, mmHg	77.5 ± 12.8	75.3 ± 13.2	80.0 ± 13.0	< 0.001	75.8 ± 11.0	77.1 ± 11.9	0.27
Heart rate, beats/min	70.4 ± 12.4	69.8 ± 13.0	71.3 ± 12.0	0.08	68.9 ± 11.8	71.0 ± 12.7	0.09
Creatinine, mg/dL	0.81 ± 0.27	0.79 ± 0.22	0.79 ± 0.27	0.71	0.86 ± 0.32	0.82 ± 0.31	0.16
Uric acid, mg/dL	5.6 ± 1.5	5.6 ± 1.5	5.7 ± 1.5	0.47	5.4 ± 1.5	5.5 ± 1.4	0.64
Total cholesterol, mg/dL	191 ± 38	168 ± 24	218 ± 32	< 0.001	162 ± 25	206 ± 34	< 0.001
Triglycerides, mg/dL	138 ± 90	135 ± 102	139 ± 88	0.47	129 ± 76	157 ± 84	< 0.001
High-density lipoprotein cholesterol, mg/dL	59 ± 17	61.3 ± 18.7	57.9 ± 15.4	0.002	59.6 ± 16.4	56.1 ± 15.5	0.04
Low-density lipoprotein cholesterol, mg/dL	107 ± 34	80.7 ± 15.1	134.4 ± 25.7	< 0.001	77.2 ± 15.9	122.8 ± 21.7	< 0.001
Glucose, mg/dL	114 ± 38	108 ± 31	112 ± 39	0.12	123 ± 41	122 ± 44	0.84
Hemoglobin A1c, %	5.6 ± 0.79	5.4 ± 0.7	5.6 ± 0.8	0.008	5.9 ± 0.8	5.8 ± 0.7	0.83
Medications, n (%)							
Calcium channel blockers	538 (40.6)	129 (33.2)	208 (38.0)	0.13	130 (53.9)	70 (47.3)	0.20
Alpha-blockers	49 (3.7)	13 (3.3)	20 (3.7)	0.79	10 (4.2)	6 (4.1)	0.96
Beta-blockers	187 (14.1)	42 (10.8)	43 (7.9)	0.12	68 (28.2)	33 (22.3)	0.20
Renin-angiotensin system inhibitors	433 (32.7)	90 (23.1)	140 (25.6)	0.39	139 (57.7)	63 (42.6)	0.004
Medically treated diabetes mellitus							
Any	218 (16.2)	39 (9.8)	40 (7.2)	0.15	92 (36.7)	47 (33.3)	0.51
Insulin-dependent	32 (2.4)	7 (1.8)	8 (1.5)	0.69	12 (5.0)	5 (3.4)	0.45
Medical history, n (%)							
Hypertension	905 (67.2)	222 (55.5)	375 (67.7)	< 0.001	198 (79.2)	108 (76.6)	0.55
Diabetes mellitus	339 (25.2)	63 (15.8)	93 (16.7)	0.69	120 (48.0)	61 (43.9)	0.44
Peripheral artery disease	121 (9.1)	33 (8.4)	39 (7.2)	0.50	34 (13.7)	14 (10.1)	0.31
Coronary artery disease	162 (12.0)	20 (5.0)	19 (3.5)	0.24	98 (39.2)	24 (17.3)	< 0.001
Cerebrovascular disease	86 (6.4)	17 (4.3)	25 (4.6)	0.83	26 (10.5)	18 (13.0)	0.46
Cardiovascular disease	223 (16.8)	36 (9.1)	39 (7.2)	0.28	110 (44.2)	38 (27.3)	0.001
Current Smoking, n (%)	268 (20.1)	119 (30.3)	99 (18.0)	< 0.001	30 (12.1)	20 (14.4)	0.51
Smoking, pack year	30.1 ± 29.7	26.7 ± 28.1	28.3 ± 27.1	0.52	41.1 ± 35.7	33.8 ± 25.5	0.13
Flow-mediated vasodilation, %	4.1 ± 3.1	4.9 ± 3.3	3.9 ± 3.0	< 0.001	3.5 ± 2.7	3.7 ± 2.8	0.56
Nitroglycerine-induced vasodilation	12.5 ± 5.7	13.7 ± 5.6	12.5 ± 5.5	0.002	11.3 ± 5.8	11.1 ± 5.9	0.72

LDL-C indicates low-density lipoprotein cholesterol.

### Relationships between LDL-C and vascular function

**Figure 1:**
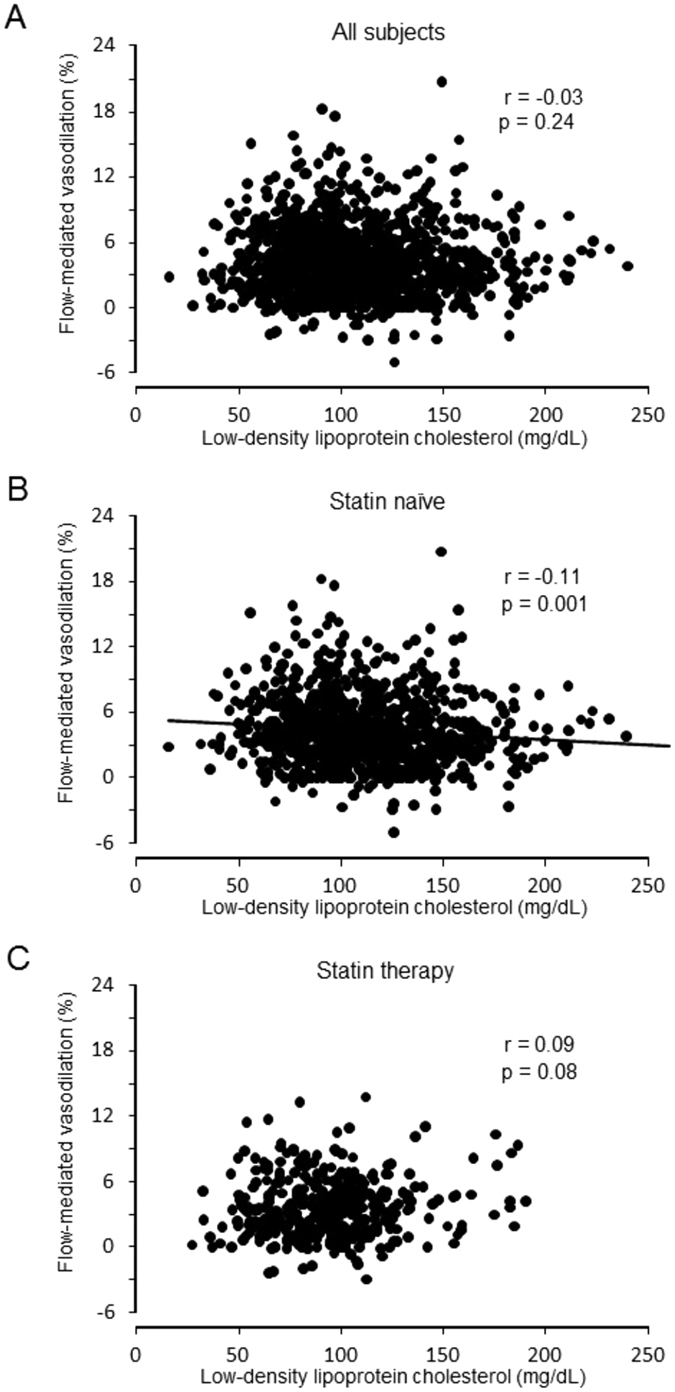
Scatter plots show the relationship between flow-mediated vasodilation and serum low-density lipoprotein cholesterol in all subjects (**A**), subjects not receiving statin therapy (**B**), and subjects receiving statin therapy (**C**).

**Figure 2:**
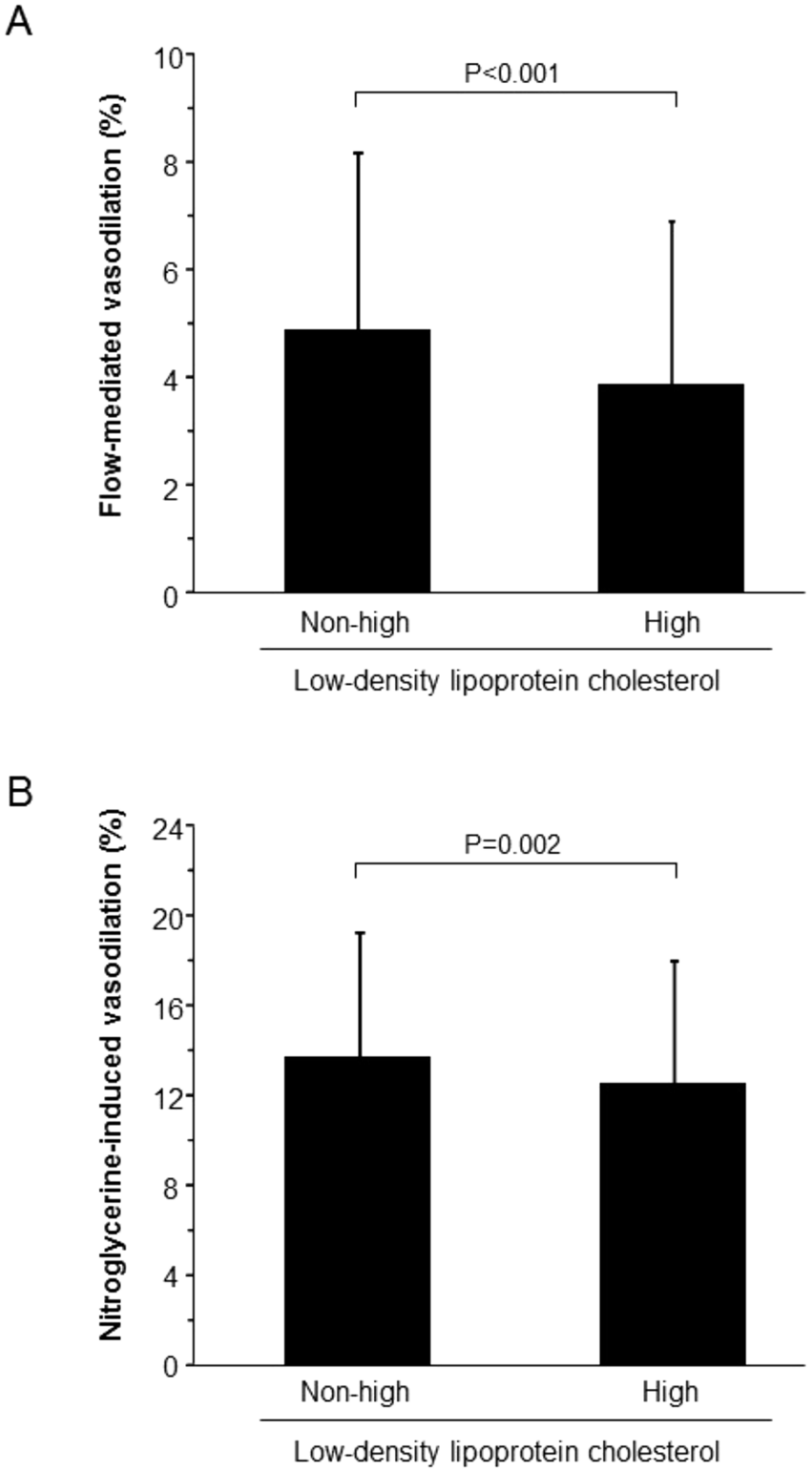
Scatter plots show the relationship between nitroglycerine-induced vasodilation and serum low-density lipoprotein cholesterol in all subjects (**A**), subjects not receiving statin therapy (**B**), and subjects receiving statin therapy (**C**).

**Figure 3:**
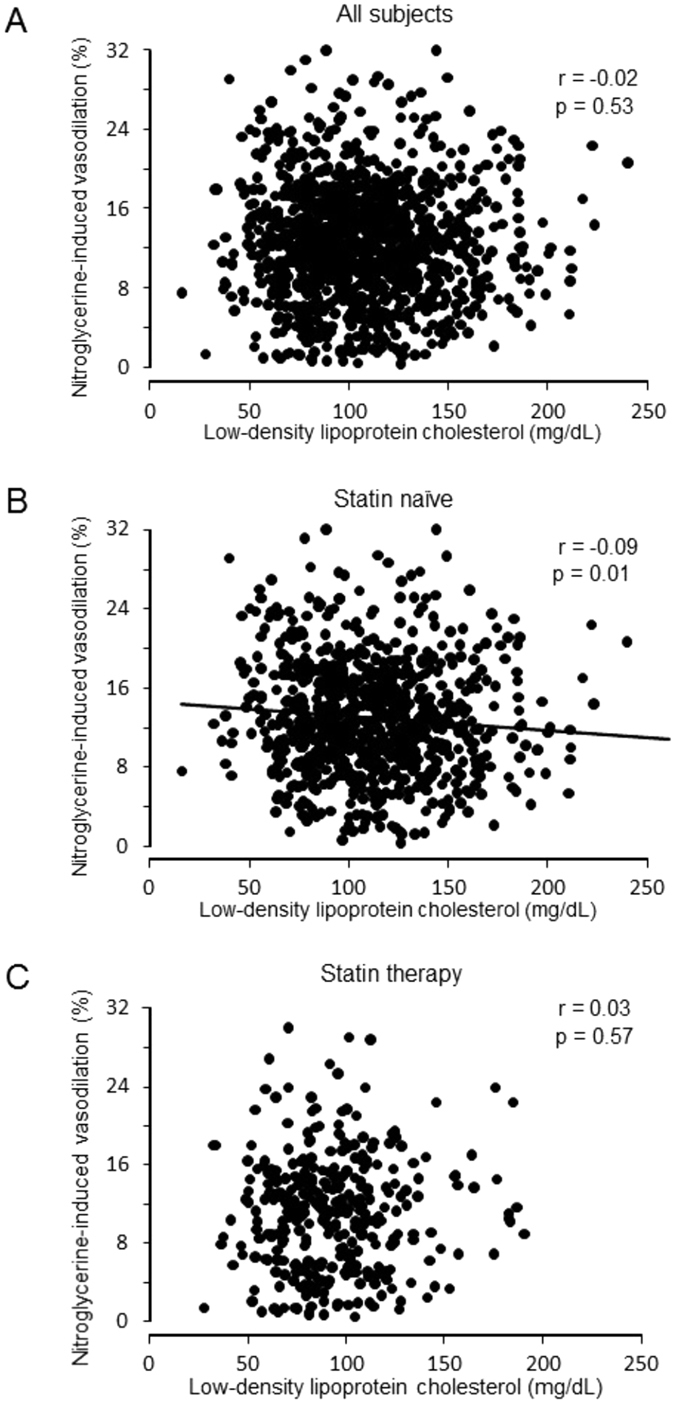
Bar graphs show flow-mediated vasodilation (**A**) and nitroglycerine-induced vasodilation (**B**) in the non-high and high low-density lipoprotein cholesterol group.

There was no significant correlation of FMD or NID with LDL-C in all subjects (Figs 1 and 2). FMD and NID were inversely correlated with LDL-C in subjects not receiving statin therapy (r = -0.11, p = 0.001 and r = -0.09 p = 0.01, respectively) but not in subjects receiving statin therapy (Figs 1 and 2). In statin naïve subjects, there were significant differences in FMD and NID between the non-high LDL-C group and high LDL-C group (4.9 ± 3.3% vs. 3.9 ± 3.0%, p < 0.001 and 13.7 ± 5.6% vs. 12.5 ± 5.5%, p = 0.002, respectively) (Fig. 3). In subjects receiving statin therapy, FMD and NID were similar in the non-high LDL-C group and high LDL-C group (3.5 ± 2.7% vs. 3.7 ± 2.8%, p = 0.56 and 11.3 ± 5.8% vs. 11.1 ± 5.9%, p = 0.72, respectively). In multiple logistic regression analysis, non-high LDL-C in the subjects not receiving statin therapy was independently associated with a low FMD tertile (OR: 0.62, 95% CI: 0.45–0.85; P = 0.003) and a low NID tertile (OR: 0.69, 95% CI: 0.50–0.96; P = 0.03) (Table 2).

**Table 2:** Multivariate Analysis of the Relations between Vascular Dysfunction and Mild Target Level of Low-Density Lipoprotein Cholesterol (≤100).

Variables	Low tertile of flow-mediated vasodilation		Low tertile of nitroglycerine-induced vasodilation	
	OR (95% CI)	P value	OR (95% CI)	P value
Model 1	0.60 (0.45–0.79)	<0.001	0.65 (0.48–0.86)	0.003
Model 2	0.62 (0.46–0.84)	0.002	0.68 (0.50–0.92)	0.01
Model 3	0.62 (0.45–0.85)	0.003	0.69 (0.50–0.96)	0.03

Low tertile of flow-mediated vasodilation indicates less than 2.7%.

Low tertile of nitroglycerine-induced vasodilation indicates less than 10.6%.

Model 1: unadjusted model.

Model 2: adjusted for age, gender.

Model 3: adjusted for age, gender, body mass index, current smoking, presence of hypertension and diabetes mellitus.

### Optimal level of LDL-C for maintenance of vascular function

**Figure 4:**
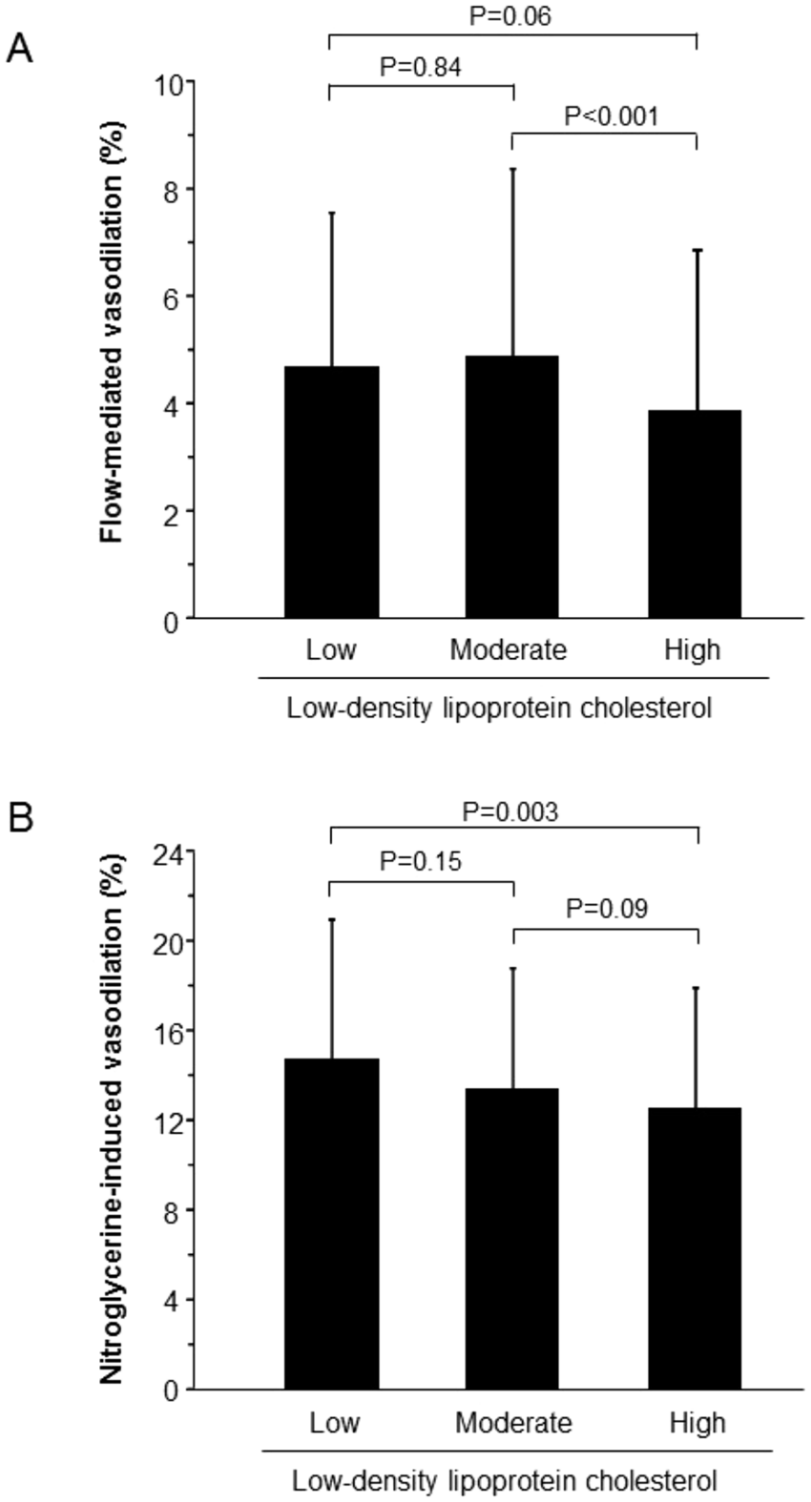
Bar graphs show flow-mediated vasodilation (**A**) and nitroglycerine-induced vasodilation (**B**) in the low, moderate and high low-density lipoprotein cholesterol group.

To determine the optimal level of LDL-C for maintenance of vascular function, we divided statin naïve subjects into three groups according to a more intensive cutoff level of LDL-C on the basis of levels recommended in current guidelines: low LDL-C group (≤70 mg/dL), moderate LDL-C group (70.1–100 mg/dL), and high LDL-C group (>100 mg/dL). Baseline clinical characteristics are summarized in Supplementary Table 2. There were significant differences in age, gender, body mass index, systolic blood pressure, diastolic blood pressure, total cholesterol, high-density lipoprotein cholesterol, low-density lipoprotein cholesterol, hemoglobin A1c and presences of hypertension, coronary artery disease and current smoking among the three groups (Supplementary Table 2). FMD was significantly lower in the high LDL-C group than in the moderate LDL-C group (3.9 ± 3.0% vs. 4.9 ± 3.5% p < 0.001) (Fig. 4). NID was significantly lower in the high LDL-C group than in the low LDL-C group (12.5 ± 5.5% vs. 14.7 ± 6.3% p = 0.003) (Fig. 4). There was no significant difference in FMD or NID between the low LDL-C group and moderate LDL-C group.

Using the high LDL-C group as a reference, low LDL-C (OR: 0.47, 95% CI, 0.27–0.81; P = 0.006) and moderate LDL-C (OR: 0.66, 95% CI, 0.48–0.94; P = 0.02) were independently associated with a decrease in the low FMD tertile in multiple logistic regression analysis (Table 3). Using the high LDL-C group as a reference, moderate LDL-C (OR: 0.67, 95% CI, 0.47–0.96; P = 0.03) was independently associated with a decrease in the low NID tertile after multiple logistic regression analysis (Table 3). Multiple logistic regression analysis revealed that there was no significant difference in the low tertile of FMD between low and moderate LDL-C levels and that there was no significant difference in the low tertile of NID between low and moderate LDL-C levels (Table 4).

**Table 3:** Multivariate Analysis of the Relations between Vascular Dysfunction and Low-Density Lipoprotein Cholesterol.

Variables	High level of low-density lipoprotein cholesterol		Low level of low-density lipoprotein cholesterol		Moderate level of low-density lipoprotein cholesterol	
	OR (95% CI)	P value	OR (95% CI)	P value	OR (95% CI)	P value
Flow-mediated vasodilation						
Model 1	1 (reference)		0.49 (0.29–0.79)	0.003	0.64 (0.47–0.86)	0.003
Model 2	1 (reference)		0.46 (0.27–0.77)	0.003	0.69 (0.49–0.95)	0.02
Model 3	1 (reference)		0.47 (0.27–0.81)	0.006	0.66 (0.48–0.94)	0.02
Nitroglycerine-induced vasodilation						
Model 1	1 (reference)		0.63 (0.38–1.03)	0.07	0.65 (0.47–0.89)	0.007
Model 2	1 (reference)		0.65 (0.38–1.08)	0.10	0.69 (0.49–0.76)	0.03
Model 3	1 (reference)		0.75 (0.43–1.29)	0.30	0.67 (0.47–0.96)	0.03

Low tertile of flow-mediated vasodilation indicates less than 2.7%.

Low tertile of nitroglycerine-induced vasodilation indicates less than 10.6%.

Model 1: unadjusted model.

Model 2: adjusted for age, gender.

Model 3: adjusted for age, gender, body mass index, current smoking, presence of hypertension and diabetes mellitus.

**Table 4:** Multivariate Analysis of the Relations between Vascular Dysfunction and Low-Density Lipoprotein Cholesterol.

Variables	Moderate level of low-density lipoprotein cholesterol		Low level of low-density lipoprotein cholesterol		High level of low-density lipoprotein cholesterol	
	OR (95% CI)	P value	OR (95% CI)	P value	OR (95% CI)	P value
Flow-mediated vasodilation						
Model 1	1 (reference)		0.76 (0.44–1.28)	0.31	1.57 (1.16–2.12)	0.003
Model 2	1 (reference)		0.67 (0.38–1.17)	0.16	1.46 (1.06–2.02)	0.02
Model 3	1 (reference)		0.70 (0.37–1.24)	0.22	1.49 (1.06–2.10)	0.02
Nitroglycerine-induced vasodilation						
Model 1	1 (reference)		0.97 (0.56–1.65)	0.72	1.54 (1.10–2.11)	0.007
Model 2	1 (reference)		0.94 (0.34–1.63)	0.83	1.45 (1.04–2.03)	0.03
Model 3	1 (reference)		0.90 (0.51–1.62)	0.72	1.48 (1.04–2.13)	0.03

Low tertile of flow-mediated vasodilation indicates less than 2.7%.

Low tertile of nitroglycerine-induced vasodilation indicates less than 10.6%.

Model 1: unadjusted model.

Model 2: adjusted for age, gender.

Model 3: adjusted for age, gender, body mass index, current smoking, presence of hypertension and diabetes mellitus.

## Discussion

The present study demonstrated that (1) vascular function assessed by FMD and NID was significantly correlated with levels of LDL-C in subjects not receiving statin therapy but not in subjects receiving statin monotherapy and that (2) vascular function in statin naïve subjects was more greatly impaired in subjects with LDL-C of >100 mg/dL than in subjects with LDL-C of ≤100 mg/dL, while an additional benefit was not observed in subjects with LDL-C of ≤70.0 mg/dL compared with subjects with LDL-C of 70.1–100 mg/dL. In the present study, we confirmed, for the first time, a significant correlation between LDL-C and vascular function even in a general population not receiving lipid-lowering therapy.

Although LDL-C is known as a strong predictor of cardiovascular events, whether a high level of LDL-C is associated with vascular dysfunction was controversial in previous studies^21, 35, 36^. Those studies included subjects receiving lipid-lowering therapy. Therefore, LDL-C-lowering therapy might affect vascular function. Recently, several studies have shown that familial hypercholesterolemia on the basis of the level of LDL-C is underdiagnosed^37^. One possible reason is inferred that cholesterol-lowering therapy hinders accurate diagnosis of familial hypercholesterolemia. In the present study, the association of LDL-C with vascular function was different in subjects receiving and those not receiving statin therapy. The inverse relationship between LDL-C and vascular function may not be evident in subjects receiving statin therapy, suggesting that stain therapy protects against adverse vascular effects of LDL-C through anti-inflammatory effects or other mechanisms.

Endothelial dysfunction is an initial step of atherosclerosis, leading to cardiovascular diseases^12, 13^. Previous studies showed that LDL-C lowered by statin therapy, but not that lowered by non-statin therapy, improved endothelial function, suggesting that statin therapy-induced improvement of endothelial function is due to pleiotropic effects, not LDL-C-lowering effects per se^38–41^. In the present study, a low level of LDL-C was beneficial for endothelial function in statin naïve subjects who had no influence of a statin. To our knowledge, this is the first report showing a correlation between LDL-C and endothelial function in statin naïve patients. Interestingly, NID, as an index of vascular smooth muscle function, was also significantly correlated with LDL-C levels in statin naïve patients. These findings support evidence indicating that non-statin lipid-lowering therapy decreases cardiovascular events, although it is established that statin therapy is beneficial for the prevention of cardiovascular events^42–44^. An interaction of Rho-associated kinase (ROCK) activity and the endothelial NO synthase (eNOS)/NO pathway plays critical roles in vascular function^45–47^. An increase in ROCK activity and a decrease in NO bioavailability independently and cooperatively impair vascular function^45–47^. It is well known that a statin inhibits ROCK activity, upregulates eNOS expression, and increases eNOS bioactivity^38, 39^. Although we do not know the precise mechanisms underlying the association between LDL-C and vascular function, an increase in ROCK activity and inactivation of NO bioavailability may contribute to vascular dysfunction in subjects with high levels of LDL-C.

There is no consensus among international guidelines on the use of a “treat-to-target” strategy with specific LDL-C goals^7–11^. Moreover, there are differences in target levels of LDL-C between the guidelines^7–10^. In most guidelines, the target LDL-C level for secondary prevention of cardiovascular events is 100 mg/dL or less. Recently, The Improved Reduction of Outcomes: Vytorin Efficacy International Trial demonstrated that in patients with acute coronary syndromes, intensive targeting of LDL-C level of 53.7 mg/dL achieved by treatment with simvastatin in combination of ezetimibe reduced cardiovascular events compared to that with LDL-C level of 69.5 mg/dL achieved by simvastatin monotherapy, suggesting that lower LDL-C levels are better for prevention of cardiovascular events in these patients^42^. In the present study, in statin naïve subjects, FMD and NID were significantly lower in subjects with LDL-C of >100 mg/dL than in subjects with LDL-C of ≤100 mg/dL. A significant difference in FMD was not observed between subjects with LDL-C of 70 to 100 mg/dL and those with LDL-C of ≤70 mg/dL. Leibowitz *et al*.^48^. showed that no additional benefit is obtained by intensive cholesterol lowering to 70 mg/dL or less for secondary prevention in patients with coronary heart disease. Their findings are consistent with our results, indicating that a “treat-to-target” strategy and a target level of LDL-C of 100 md/dL are optimal for maintenance of vascular function. Recently, the Further Cardiovascular Outcomes Research with PCSK9 Inhibition in Subjects with Elevated Risk trial has shown that inhibition of PCSK9 in combination with statin therapy lowers LDL cholesterol level from 70 mg/dL to 30 mg/dL and reduces cardiovascular outcomes in patients with established cardiovascular disease^49^. These findings suggest that LDL cholesterol-lowering therapy that is more intensive than the current moderate-intensity LDL cholesterol-lowering therapy with a statin would be more effective for prevention of cardiovascular events. From the aspect of vascular function also, low levels of LDL cholesterol of 30 mg/dL or less may be beneficial for endothelial function. In the present study, few subjects had LDL cholesterol levels of 30 mg/dL or less. Future studies are needed to evaluate the effects of more intensive LDL cholesterol-lowering therapy on vascular function in a large population trial.

There were several limitations in the present study. First, the cross-sectional design did not allow us to establish a definitive causal relationship between LDL-C and vascular function. Future prospective and interventional studies are needed to establish more specific conclusions as to whether elevation of serum levels of LDL-C is associated with vascular dysfunction.

Second, in the present study, a significant correlation was not found between LDL-C and FMD in subjects receiving statin monotherapy, while a low level of LDL-C achieved by cholesterol-lowering therapy has been established to be essential for prevention of cardiovascular disease. In the subjects receiving statin monotherapy, the percentage of subjects who had a history of previous cardiovascular diseases progressively increased from the high LDL-C group to the low LDL-C group (Supplementary Table 2). Thus, we cannot rule out the possibility that there were differences in baseline characteristics among the groups. In addition, the intensity of the statin per se and dosages of statins were not considered. It is necessary to adjust baseline characteristics and the intensity, treatment period, and dosage of statins for assessment of vascular function in patients receiving statin therapy. Even after adjustment of these parameters, it would be difficult to assess vascular function under the condition of statin treatment.

In conclusion, LDL-C is an independent risk factor for vascular dysfunction in statin naïve individuals but not in individuals receiving statin monotherapy. In a general population not receiving lipid-lowering therapy, LDL-C of 100 mg/dL may be optimal as a cutoff level for maintenance of vascular function.

## Methods

### Subjects

A total of 1349 subjects were recruited from people who underwent a health check up at Hiroshima University Hospital between August 2007 and August 2016. Subjects were excluded if they were newly prescribed a statin or changed statin doses within 6 months. In order to focus on statin user and statin naïve patients, subjects were also excluded if they were receiving non-statin therapy: ezetimibe, PCSK9 inhibitor, bile acid sequestrants, fibrates, and niacin. Patients with hypertension were defined as those taking oral antihypertensive agents or with systolic blood pressure of more than 140 mm Hg or diastolic blood pressure of more than 90 mm Hg measured in a sitting position on at least 3 different occasions. Diabetes mellitus was defined according to the American Diabetes Association recommendation^50^. Dyslipidemia was defined according to the third report of the National Cholesterol Education Program^51^. Smokers were defined as those who were current smokers. One pack-year was equivalent to 20 cigarettes per day for 1 year. Coronary heart disease included angina pectoris, myocardial infarction, and unstable angina. Cerebrovascular disease included ischemic stroke, hemorrhagic stroke, and transient ischemic attack. Cardiovascular disease was defined as coronary heart disease and cerebrovascular disease. LDL-C concentration was calculated directly for subjects with triglycerides of >400 mg/dL. The Friedewald equation was used for other subjects.

In a primary analysis, we defined the target level of LDL-C as 100 mg/dL and divided the subjects into the two groups: a non-high group (LDL-C of ≤100 mg/dL) and a high group (LDL-C of >100 mg/dL). In a secondary analysis, the subjects were divided into the three groups to investigate whether an additional benefit is achieved in the lower level of LDL-C: a low group (LDL-C of ≤70 mg/dL), moderate group (LDL-C of 70.1–100 mg/dL), and high group (LDL-C of >100 mg/dL).

All methods were carried out in accordance with relevant guidelines and regulations. The Ethics Review Board of Hiroshima University approved the study protocol. Written informed consent for participation in the study was obtained from all of the subjects. All methods were performed in accordance with the relevant guidelines and regulations overseen by the Ethical Committee.

### Study protocol

All subjects were assessed vascular function and structure using measurement of FMD and NID in brachial artery. The subjects fasted overnight for at least 12 hours and the study began at 08:30 hours, and remained supine in a quiet, dark, air-conditioned room (constant temperature of 22 °C to 25 °C) throughout the study. A 23-gauge polyethylene catheter was inserted into the left deep antecubital vein to obtain blood samples. At 30 minutes of maintaining a supine position, FMD and NID were measured. The observers were blind to the form of examination.

### Measurements of FMD and NID

We evaluated the vascular response to reactive hyperemia in the brachial artery for assessment of endothelium-dependent FMD. A high resolution linear artery transducer was coupled to computer assisted analysis software (UNEX18 G, UNEX Co, Nagoya, Japan) that used an automated edge detection system for measurement of brachial artery diameter. A blood pressure cuff was placed around the forearm. The brachial artery was scanned longitudinally 5–10 cm above the elbow. When the clearest B-mode image of the anterior and posterior intimal interfaces between the lumen and vessel wall was obtained, the transducer was held at the same point throughout the scan by a special probe holder (UNEX Co) to ensure consistency of the image. Depth and gain settings were set to optimize the images of the arterial lumen wall interface. When the tracking gate was placed on the intima, the artery diameter was automatically tracked, and the waveform of diameter changes over the cardiac cycle was displayed in real time using the FMD mode of the tracking system. This allowed the ultrasound images to be optimized at the start of the scan and the transducer position to be adjusted immediately for optimal tracking performance throughout the scan. Pulsed Doppler flow was assessed at baseline and during peak hyperemic flow, which was confirmed to occur within 15 seconds after cuff deflation. Blood flow velocity was calculated from the Doppler data and was displayed as a waveform in real time. The baseline longitudinal image of the artery was acquired for 30 seconds, and then the blood pressure cuff was inflated to 50 mm Hg above systolic pressure for 5 minutes. The longitudinal images of the artery was recorded continuously until 5 minutes after cuff deflation. Pulsed Doppler velocity signals were obtained for 20 seconds at baseline and for 10 seconds immediately after cuff deflation. Changes in brachial artery diameter were immediately expressed as the percentage change relative to the vessel diameter before cuff inflation. FMD was automatically calculated as the percentage change in peak vessel diameter from the baseline value. Percentage of FMD [(Peak diameter–Base line diameter)/Baseline diameter] was used for analysis. Blood flow volume was calculated by multiplying the Doppler flow velocity (corrected for the angle) by the heart rate and vessel cross-sectional area (−r^2^). Reactive hyperemia was calculated as the maximum percentage increase in flow after cuff deflation compared with baseline flow.

The response to nitroglycerine was used for assessment of endothelium-independent vasodilation^33^. After acquiring baseline rest image for 30 seconds, a sublingual tablet (nitroglycerine 75 μg) was given and imaging of the artery was done continuously for 5 minutes. NID was automatically calculated as a percentage change in peak vessel diameter from the baseline. Percentage of NID [(Peak diameter–Baseline diameter)/Baseline diameter] was used for analysis. In our laboratory, the coefficient of variation for the baseline diameter was 2.9%.

### Statistical analysis

Results are presented as means ± SD. All reported probability values were 2-sided, and a probability value of <0.05 was considered statistically significant. Categorical values were compared by means of the chi-square test. Continuous variables were compared by using ANOVA multiple groups. Comparisons between the groups categorized according to the serum LDL-C were carried out using repeated measures ANOVA with tukey’s post hoc test. Multiple logistic regression analysis was performed to identify independent variables associated with low tertiles of FMD (<2.7%) and NID (<10.6%). Age, sex, body mass index, and presences of current smoking, hypertension and diabetes mellitus were entered into the multiple logistic regression analysis. The data were processed using the software package Stata version 9 (Stata Co. College Station, Texas, USA).

### Clinical Trial Registration Information

URL for Clinical Trial: http://UMIN; Registration Number for Clinical Trial: UMIN000003409.

## Electronic supplementary material

Supplementary information
