# Supplementary material for: Optimal Target Level of Low-density Lipoprotein Cholesterol for Vascular Function in Statin Naïve Individuals
Source: Sci Rep. 2017 Aug 21;7:8422. doi: 10.1038/s41598-017-09043-1 (PMC5566450; doi:10.1038/s41598-017-09043-1)
Supplement: Supplementary file 1 — Supplementary information [file 41598_2017_9043_MOESM1_ESM.pdf]

Supplementary information

Optimal Target Level of Low-Density Lipoprotein Cholesterol for Vascular Function in Statin Naïve Individuals

Shogo Matsui, MD;[1] Masato Kajikawa, MD, PhD;[2] Eisuke Hida, PhD;[3] Tatsuya Maruhashi, MD, PhD;[1] Yumiko Iwamoto, MD;[1] Akimichi Iwamoto, MD;[1] Nozomu Oda, MD;[1] Shinji Kishimoto, MD;[1] Takayuki Hidaka, MD, PhD;[1] Yasuki Kihara, MD, PhD;[1] Kazuaki Chayama, MD, PhD;[4] Chikara Goto, PhD;[5] Yoshiki Aibara, MS;[6] Ayumu Nakashima, MD, PhD;[6] Farina Binti Mohamad Yusoff, MD;[6] Kensuke Noma, MD, PhD;[2,6]
Yukihito Higashi, MD, PhD, FAHA[2,6]

[1]Department of Cardiovascular Medicine, Hiroshima University Graduate School of Biomedical Sciences, Hiroshima, Japan
[2]Division of Regeneration and Medicine, Medical Center for Translational and Clinical Research, Hiroshima University Hospital, Hiroshima, Japan
[3]Center for Integrated Medical Research, Hiroshima University Hospital, Hiroshima, Japan
[4]Department of Gastroenterology and Metabolism, Institute of Biomedical and Health Sciences, Graduate School of Biomedical and Health Sciences, Hiroshima University Hiroshima, Japan
[5]Hiroshima International University, Hiroshima, Japan
[6]Department of Cardiovascular Regeneration and Medicine, Research Institute for Radiation Biology and Medicine, Hiroshima University, Hiroshima, Japan

Address for correspondence: Yukihito Higashi, MD, PhD, FAHA
Department of Cardiovascular Regeneration and Medicine,
Research Institute for Radiation Biology and Medicine (RIRBM), Hiroshima University
1-2-3 Kasumi, Minami-ku, Hiroshima 734-8551, Japan
Phone: +81-82-257-5831 Fax: +81-82-257-5831
E-mail: yhigashi@hiroshima-u.ac.jp

Supplemental Tables

Table S1. Univariate Analysis of the Relations between Vascular Function and Variables

Variables	All subjects				Subjects not receiving statin therapy				Subjects receiving statin therapy			
	Flow-mediated vasodilation		Nitroglycerine-induced vasodilation		Flow-mediated vasodilation		Nitroglycerine-induced vasodilation		Flow-mediated vasodilation		Nitroglycerine-induced vasodilation	
	β	P value	β	P value	β	P value	β	P value	β	P value	β	P value
Age, y	-0.37	<0.001	-0.33	<0.001	-0.40	<0.001	-0.33	<0.001	-0.17	<0.001	-0.26	<0.001
Body mass index, kg/m^2	-0.07	0.01	-0.08	0.006	-0.09	0.007	-0.13	<0.001	0.03	0.53	0.08	0.16
Systolic blood pressure, mmHg	-0.16	<0.001	-0.18	<0.001	-0.19	<0.001	-0.20	<0.001	-0.05	0.35	-0.11	0.04
Diastolic blood pressure, mmHg	-0.13	<0.001	-0.08	0.006	-0.19	<0.001	-0.15	<0.001	-0.008	0.88	-0.06	0.26
Total cholesterol, mg/dl	-0.06	0.048	0.01	0.63	-0.14	<0.001	0.08	0.03	0.09	0.09	0.11	0.05
Triglycerides, mg/dL	-0.11	<0.001	-0.01	0.79	-0.14	<0.001	-0.04	0.26	0.002	0.97	0.09	0.11
High-density lipoprotein cholesterol, mg/dL	0.01	0.82	0.01	0.73	0.02	0.63	0.004	0.92	-0.04	0.48	0.01	0.88
Low-density lipoprotein cholesterol, mg/dL	-0.03	0.24	-0.02	0.53	-0.11	0.001	-0.09	0.01	-0.09	0.08	0.03	0.57

Glucose, mg/dL	-0.13	<0.001	-0.13	<0.001	-0.14	<0.001	-0.14	<0.001	-0.06	0.29	-0.05	0.35
Hemoglobin A1c, %	-0.15	<0.001	-0.19	<0.001	-0.17	<0.001	-0.17	<0.001	-0.03	0.62	-0.11	0.07
Pack Years	-0.21	<0.001	-0.14	<0.001	-0.27	<0.001	-0.16	<0.001	-0.05	0.44	-0.06	0.42

Table S2. Clinical Characteristics of the Subjects in Accordance with LDL-C

Variables	Subjects not receiving statin therapy			P value	Subjects receiving statin therapy			P value
	LDL-C ≤70 (n=96)	70< LDL-C ≤100 (n=304)	100< LDL-C (557)		LDL-C ≤70 (n=88)	70< LDL-C ≤100 (n=163)	100< LDL-C (n=141)	
Age, yr	53±20	52±20	57±16	<0.001	68±11	69±8	67±11	0.18
Gender, men/women	73/23	206/98	306/251	<0.001	61/27	79/84	62/79	<0.001
Body mass index, kg/m^2	22.3±4.0	22.7±3.5	24.1±4.1	<0.001	24.9±5.0	24.1±3.4	24.3±3.2	0.28
Systolic blood pressure, mmHg	126.3±20.6	127.1±19.3	133.6±19.8	<0.001	134.4±21.2	131.6±18.2	132.8±18.0	0.52
Diastolic blood pressure, mmHg	75.4±13.8	75.2±13.0	80.0±13.0	<0.001	74.8±11.9	76.3±10.4	77.1±11.9	0.32
Heart rate, beats/min	69±13	70±13	71±12	0.18	69±12	69±12	71±13	0.23
Creatinine, mg/dL	0.81±0.19	0.79±0.23	0.79±0.27	0.79	0.93±0.37	0.82±0.28	0.82±0.31	0.02
Uric acid, mg/dL	5.7±1.5	5.6±1.5	5.7±1.5	0.56	5.4±1.3	5.4±1.6	5.5±1.4	0.85
Total cholesterol, mg/dL	149±23	174±21	218±32	<0.001	142±21	172±20	206±33	<0.001
Triglycerides, mg/dL	145±117	132±97	139±88	0.37	133±91	126±66	157±84	0.003
High-density lipoprotein cholesterol, mg/dL	63±20	61±18	58±15	0.004	57±17	61±16	56±15	0.04
Low-density lipoprotein cholesterol, mg/dL	59±10	87±9	134±26	<0.001	59±10	87±9	123±22	<0.001

Glucose, mg/dL	114±36	106±29	111±39	0.08	124±45	122±39	122±44	0.87
Hemoglobin A1c, %	5.4±0.78	5.4±0.67	5.6±0.82	0.03	5.9±0.79	5.8±0.77	5.8±0.73	0.78
Medications, n (%)								
Calcium channel blockers	21 (22.8)	104 (36.4)	212 (38.0)	0.02	44 (51.2)	88 (54.0)	68 (48.6)	0.64
Alpha-blockers	4 (4.4)	9 (3.0)	20 (3.7)	0.81	3 (3.5)	7 (4.3)	6 (4.3)	0.95
Beta-blockers	15 (16.3)	27 (9.1)	43 (7.9)	0.03	31 (36.1)	40 (24.5)	30 (21.4)	0.04
Renin-angiotensin system inhibitors	21 (22.8)	69 (23.2)	140 (25.6)	0.69	58 (67.4)	85 (52.2)	59 (42.1)	0.001
Medically treated diabetes mellitus								
Any	13 (13.5)	26 (8.6)	40 (7.2)	0.11	40 (45.5)	52 (31.9)	47 (33.3)	0.08
Insulin-dependent	1 (1.1)	6 (2.0)	8 (1.5)	0.76	5 (5.81)	7 (4.3)	5 (3.6)	0.72
Medical history, n (%)								
Hypertension	48 (50.0)	174 (57.2)	375 (67.7)	<0.001	73 (83.0)	126 (77.3)	108 (76.6)	0.48
Diabetes mellitus	18 (18.8)	45 (14.8)	93 (16.7)	0.61	46 (52.3)	75 (46.0)	61 (43.9)	0.46
Peripheral artery disease	9 (9.6)	24 (8.0)	39 (7.2)	0.70	22 (25.3)	13 (8.0)	14 (10.1)	<0.001
Coronary artery disease	10 (10.5)	10 (3.3)	19 (3.5)	0.004	45 (51.1)	53 (32.5)	24 (17.3)	<0.001
Cerebrovascular disease	2 (2.1)	15 (5.0)	25 (4.6)	0.49	13 (14.9)	13 (8.0)	18 (13.0)	0.20
Cardiovascular disease	12 (12.8)	24 (8.0)	39 (7.2)	0.18	50 (57.5)	60 (37.0)	38 (27.3)	<0.001
Current Smoking, n (%)	34 (35.4)	85 (28.6)	99 (18.0)	<0.001	14 (16.1)	16 (9.8)	20 (14.4)	0.29
Smoking, pack year	30.8±31.3	25.5±27.0	28.3±27.1	0.37	43.2±35.9	39.1±35.6	33.9±25.1	0.27

Flow-mediated vasodilation, %	4.7±2.9	4.9±3.5	3.9±3.0	<0.001	3.5±2.8	3.5±2.7	3.7±2.8	0.87
Nitroglycerine-induced vasodilation	14.7±6.3	13.4±5.4	12.5±5.5	0.002	11.6±6.0	11.1±5.7	11.1±5.9	0.80

LDL-C indicates Low-density lipoprotein cholesterol.
